# Proportion of Maternal Near-Miss and Its Determinants among Northwest Ethiopian Women: A Cross-Sectional Study

**DOI:** 10.1155/2020/5257431

**Published:** 2020-03-18

**Authors:** Mengstu Melkamu Asaye

**Affiliations:** Women and Family Health Department, School of Midwifery, College of Medicine and Health Sciences, University of Gondar, P.O. Box 196, Ethiopia

## Abstract

**Background:**

Life-threatening situations might arise unexpectedly during pregnancy. Maternal near-miss can be a proxy for maternal death and explained as women who nearly died due to obstetric-related complications. It is recognized as the predictor of level of care and maternal death. Maternal near-miss evaluates life-threatening pregnancy-related complications, and it directs the assessment of the quality of obstetric care.

**Objective:**

To determine the proportion and factors associated with maternal near-miss at maternity wards at the University of Gondar Referral Hospital, Northwest Ethiopia, 2019.

**Methods:**

A cross-sectional study design was carried out from March 1 to June 20, 2019, using WHO criteria for maternal near-miss at the University of Gondar Referral Hospital. The data are from the interviews and review of 303 systematically selected participants' medical files at maternity wards. Bivariate and multivariable logistic regression analyses were performed to analyze factors associated with maternal near-miss, including estimation of crude and adjusted odds ratios and their respective 95% confidence intervals and *p* value less than 0.05 through SPSS version 20.

**Result:**

The study revealed that the proportion of maternal near-miss was found to be 15.8% (95%CI = 11.9%-20.1%). In the adjusted analyses, maternal near-miss was significantly associated with low (≤1000 ETB) monthly income (AOR = 399; 95%CI = 1.65, 9.65), seven or more days of hospital stay (AOR = 5.43; 95%CI = 2.49, 11.83), vaginal bleeding (AOR = 2.75, 95%CI = 1.17, 6.47), and pregnancy-induced hypertension (AOR = 5.13; 95%CI = 2.08, 12.6). *Conclusion and Recommendation*. The near-miss proportion was comparable to that in the region. Associated factors were low monthly income, seven or more days of hospital stay, vaginal bleeding, and pregnancy-induced hypertension. Thus, giving attention on early identification and treatment of these potential factors can be the opportunity in the reduction of maternal morbidity and mortality.

## 1. Introduction

Maternal near-miss (MNM) refers to a situation where a women who nearly died but survived from a comprehensive range of life-threatening obstetric complication [1] that occurred during pregnancy, child birth, or within 42 days of termination of pregnancy [2].

According to WHO criteria, maternal near-miss is a sign of organ dysfunction that follow life-threatening conditions and it enables the assessment of the quality of care provided to pregnant women [3]. The inclusion criteria for maternal near-miss are categorized in three areas: clinical, laboratory, and management-based criteria. The reason is that these identification criteria may be used in a comparable way across the settings and over time. It is important to understand the process of care that the patient has undergone in order to improve the quality of care, and its applicability depends on the local context [4].

Developing countries account for approximately 99% (302,000) of global maternal deaths, and Sub-Saharan Africa alone accounts for roughly 66% (201 000) to this death [5]. The magnitude also varies among different countries and regions depending on the health care quality and availability [6]. The number of maternal death is high worldwide. This death might be associated with inadequate and poor quality of health care services. Thus, identifying the potential factors that leads to severe maternal life-threatening complications is necessary [7].

We can prevent maternal death through utilization of the near-miss data. Thus, the mother that survives a near-miss can provide more comprehensive information when reviewing maternal death cases. So it is a better predicate for preventive planning [8], and it is a useful means to improve the quality of obstetric care especially in low-income countries.

In developing countries, about 75% of the women were in a critical condition upon arrival. Availability, accessibility, cost of health care, and behavioral factors play an important role in the utilization of maternal health services [2]. Maternal near-miss events were associated with postpartum hemorrhage, sever preeclampsia, eclampsia, sepsis or severe systematic infection, ruptured uterus, and septic abortion [9, 10]. But in the current study, the main approaches to the reduction of maternal near-miss and maternal deaths are emergency obstetric care, skilled care by skilled birth attendants, and unmet obstetric need [2]. Studying maternal near-miss allows the assessment of quality interventions, identifies the health system failures, and provides alternative strategies to reduce maternal mortality [11]. Delays in initiating timely and adequate treatment for obstetric complications were a major factor to develop maternal near-miss [12, 13].

The pregnancy-related mortality ratio, in Ethiopia, is estimated to be approximately 412 death/100,000 live birth according to 2016 EDH [14]. One target of SDG is to achieve a reduction of global maternal mortality ratio to less than 70 per 100,000 live births by 2030 [15]. To achieve this goal and solve the problem, respectively, we have to review both maternal mortality and maternal near-miss cases. The present study is aimed at assessing the proportion of maternal near-miss and factors associated with it at Gondar Tertiary Hospital.

## 2. Methods

### 2.1. Study Design and Period

An institution-based cross-sectional study design was employed at the University of Gondar Referral Hospital from March 1 to June 20, 2019.

### 2.2. Setting

The study was conducted at the University of Gondar Referral Hospital, maternity wards. It is located in the North Gondar administrative zone, in Amhara region, 727 km Northwest of Addis Ababa and 175 km far from Bahirdar. Currently, Gondar town has one tertiary hospital and five health centers. The hospital provides service for 5-7 million people of the north Gondar neighboring zones. The average annual birth rate at the University of Gondar Referral Hospital is 9600 deliveries with 800 births each month.

### 2.3. Source Population

All pregnant women who were in labor, delivered or aborted, or within 42 days of postpartum period and admitted in maternity wards at Gondar Referral Hospital.

### 2.4. Inclusion Criteria

All pregnant women who came to the hospital due to early complication of pregnancy including abortion, ectopic pregnancy, and molar pregnancy and patients presenting care in labor, delivery, or postnatal units within 42 days of delivery were included.

### 2.5. Exclusion Criteria

Women with obstetric near-miss complications due to incidental or accidental causes and those who were revisiting (within data collection time) and unable to respond or seriously ill at interview time were excluded.

### 2.6. Sample Size Determination

The sample size was calculated by using the following assumptions.

Z*α*/2 = confidence level at 95% = 1.96, margin of error (*d*) = 5%, and prevalence of maternal near − miss = 23.3% [11]

*n* = (Z*α*/2)^2^(1–*p*)/*d*^2^ = (1.96)(1.96)(0.233)(0.767)/(0.05)(0.05) = 275 and 10% nonresponse rate; the final sample size was 303 women

### 2.7. Sampling Procedure

The calculated sample size was proportionally allocated to each four maternity wards based on the previous consecutive five-month average monthly client flow of the units which were obtained by referring client registration log books. The average monthly client flow for maternity 1, 2, and 3 and gynecology ward were 800, 500, 310, and 400, respectively. A total of 2010 women were booked. The study participants were selected by using a systematic random sampling technique among women who visited Gondar Tertiary Hospital during the data collection period. The first study participant was selected by a lottery method. I compute *K*^th^ for each ward, and it ranges from 1.2 to 2.6; then, every 3^rd^ women were selected during the study period.

### 2.8. Operational Definitions

*Maternal near-miss*: a woman who nearly died but survived a complication that occurred during pregnancy, childbirth, or within 42 days of termination of pregnancy according to WHO criteria (if the woman full fill one criteria) [3].

*Maternal mortality*: death of a woman while pregnant or within 42 days of termination of pregnancy, irrespective of the duration and the site of the pregnancy, from any cause related to or aggravated by the pregnancy or its management, but not from accidental or incidental causes [16].

*Delay of the decision to seek care*: The time taken greater than one hour in deciding to seek care after the onset of labor.

*Delay of reaching health facility*: a woman is unable to arrive within one hour of travelling to reach the health facility by foot.

*Delay of receiving appropriate care*: a woman did not get an emergency obstetric care within the first five minutes of arrival to health facility.

*Delays to intuitional delivery*: at least one or more delay from the three delay model [17].

*Income*: refers to total household income.

### 2.9. Data Collection Tools and Procedures

The data was collected by using semistructured interviewer-administrated questionnaires and patient charts. Questionnaires were developed in English. For interview purpose, questionnaires were translated first into Amharic and then back to English for its consistency. Four midwifery students in each ward were allocated for data collection purpose. Participants were interviewed by the data collectors after completion of examination.

### 2.10. Data Quality Control

The quality of data was assured by proper designing of the questionnaires. Half day training was given for data collectors. Every day after data collection, questionnaires were reviewed and checked for completeness by the data collector and principal investigator.

### 2.11. Data Processing and Analysis

All the questionnaires were checked, entered to Epi-info 7, and coded into SPSS version 20 software package for analysis. The degree of association between independent and dependent variables was assessed using odds ratio with 95% confidence interval. The results were presented in the form of tables, figures, and text using frequencies, and summary statistics such as percentage and logistic regression was used.

## 3. Results

A total of 303 women have responded to the questionnaires making a response rate of 100%. The age of the study participants was between 15 and 50 years with mean age of 26.41 ± 5.27 years. The majority of the participants (88.8%) were orthodox Christians, 98% of them were married, and nearly half of the study participants (48.2%) had never attended formal education.

Among the participants, 225 (74.3%) were house wives, 114 (38.4%) of their husbands were farmers in occupation, and 33% of the study participants' monthly incomes were less than or equal to 1000 EBR birr (Table 1).

### 3.1. Past Obstetric-Related Characteristics of Study Participants

**Figure 1:**
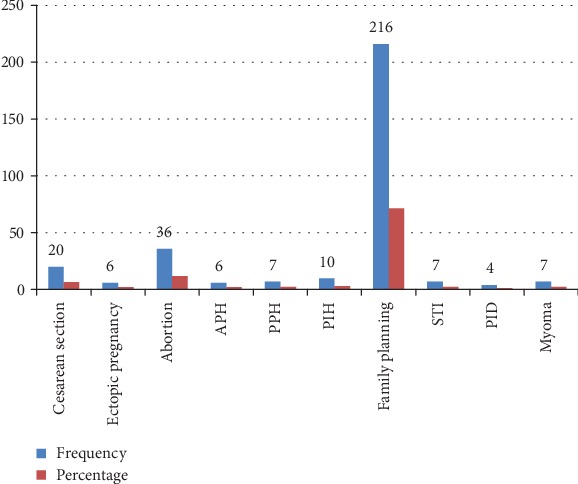
Past obstetric-related characteristics respondents at the University of Gondar Referral Hospital, Northwest Ethiopia, June 2019.

Among the 303 study participants, 6.6% had previous history of caesarean section and 3.0%, 2%, and 3.3% had previous history of APH, PPH, and PIH, respectively. Nearly three-fourth of the study participants, 65.7%, had family planning utilization history, and 11.9% of them had abortion history (Figure 1).

### 3.2. Current Obstetric-Related Characteristics of Study Participants

Nearly half, 149 (49.2%), of the study participants had two to four children while 121 (39.9%) of them had only one child. Among the study participants, 267 (88.1%) of mothers had attended antenatal care and 253 (83.5%) of the gestational age were term.

### 3.3. Maternal Health Service-Related Characteristics

**Figure 2:**
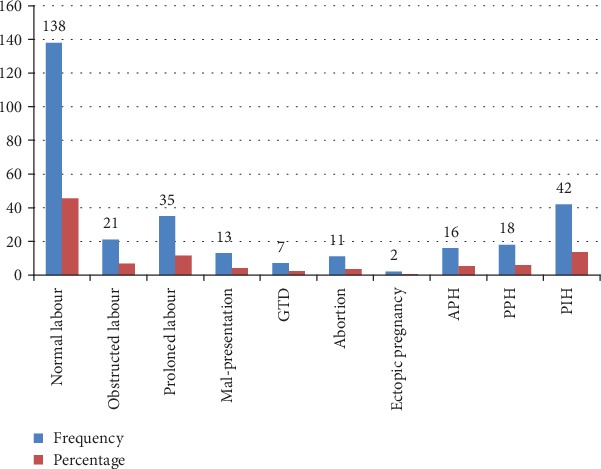
Initial admission diagnosis characteristics of respondents at the University of Gondar Referral Hospital, Northwest Ethiopia, June, 2019 (*n* = 303).

Nearly half of the women, 50.8%, had been referred. The majority, 96.4%, of the study participants were tested for hematocrit, and 72.79% of them had greater than 33%. The suggested reasons why they seek the services were 36.6% due to labor pain, 13.5% due to prolonged labor, and 61 (20.1%) of the respondents were due to vaginal bleeding. On the other hand, 45.5%, 6.9%, and 13.8% of the respondents were admitted with an initial diagnosed of normal labor, obstructed labor, and pregnancy-induced hypertension, respectively (Figure 2).

**Figure 3:**
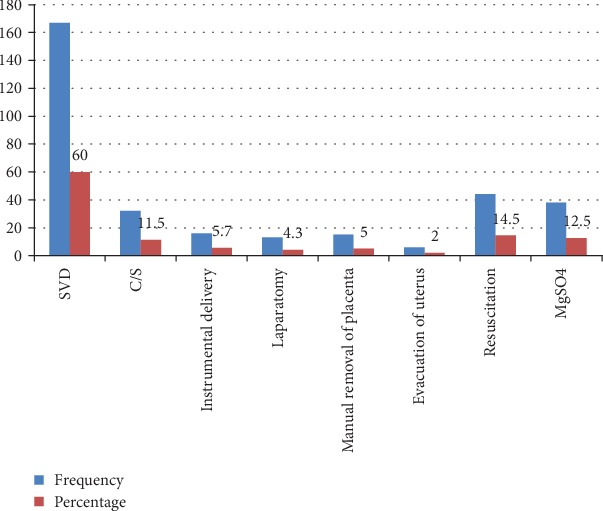
Type of intervention that was given to respondents at the University of Gondar Referral Hospital, Northwest Ethiopia, June, 2019 (*n* = 303).

One hundred sixty-seven (60%) women had spontaneous vaginal deliveries. On the other hand, 11.5% of study participants delivered by cesarean section. In addition to this, 12.5% of study participants were treated with magnesium sulphate (MgSO4) (Figure 3).

### 3.4. Three Delay Model-Related Variables of Respondents

Two hundred two study participants (80%) came to the hospital by their own decision. Delay in decision to seek care occurred in 48 (15.8%) women. The most common reason why they were delayed to reach the hospital was 16 (61.53%) due to lack of transport. One hundred sixty-seven (55.1%) respondents reported transport greater than or equal to one hour to reach the health facility (Table 2).

### 3.5. Proportion of Maternal Near-Miss

The present study revealed that the estimated proportion of maternal near-miss was 15.8% (95%CI = 11.9%-20.1%) by WHO criteria.

### 3.6. Factor Associated with Maternal Near-Miss

In bivariate binary logistic regression, residence, income, parity, duration of hospital stay, admission mode, visual disturbance, severe headache, vaginal bleeding, and pregnancy-induced hypertension (PIH) were directly associated with maternal near-miss. However, in the adjusted analyses, monthly income, duration of hospital stay, vaginal bleeding, and pregnancy-induced hypertension factors were significantly associated with maternal near-miss.

In this study, the odds of developing maternal near-miss among women who had lowest (≤1000 ETB) monthly income were 3.99 times than those who had greater than or equal to 3001 ETB per month (AOR = 3.99, 95%CI = 1.65-9.65).

In the current study, the odds of developing maternal near-miss among women who stayed seven and more days in hospital were 5.43 than those women who stayed less than seven days in the hospital (AOR = 5.43, 95%CI = 2.49-11.83). The study also suggested that women who presented with vaginal bleeding were 2.75 times more likely to develop maternal near-miss as compared to those who did not have vaginal bleeding (AOR = 2.75, 95%CI = 1.17-6.47). Lastly, the analysis showed that the odds of developing maternal near-miss among women with pregnancy-induced hypertension were 5.13 than those who did not have PIH (AOR = 5.13, 95%CI = 2.08-12.6) (Table 3).

## 4. Discussion

The aim of this study was to determine the proportion and factors associated with maternal near-miss at the University of Gondar Referral Hospital in 2019.

The present study revealed that the proportion of maternal near-miss at the University of Gondar Referral Hospital was 15.8% (95%CI = 11.9%-20.1%). This finding is comparable with a study conducted in Nigeria 17.4% [6] and Morocco 12% [18]. This similarity might be somewhat similar with sociodemographic characteristics of study participants. However, it is higher than a study done in Kerala 9.27% [13]; Harare, Zimbabwe 9.3% [19]; and Brazil 10.2% [20]. The variation might be as a result of the fact that the study institution is a tertiary hospital that receives complicated cases from remote primary hospitals and health centers within the zone and surrounding zones of the region. The second possible reason might be that in this study, 37.3% of the participants were from rural communities. This might be also correlated with delay of life-saving interventions which may impact maternal near-miss. Moreover, the last reason might be that, because of the limited facilities at the private hospitals, women with complicated pregnancies are not usually referred to these health institutions.

In contrast, this study is lower as compared with the studies conducted in South Sudan 94.1% [21], Northeast Region of Brazil 31.5% [22], Southeast Iran 25.2% [8], Brazilian demographic health survey 21.1% [23], and Debre-Markos hospital, Ethiopia, 29.7% [23]. The primary possible explanation might be due to the small sample size and short study period in the current study. The second reason might be due to the implementation of currently endorsed nonpneumatic antishock garment for postpartum hemorrhage management at the University of Gondar Referral Hospital. Lastly, it could be due to the availability of magnesium sulphate for prevention of eclampsia and broad spectrum antibiotics for the prevention of sepsis at the University of Gondar Referral Hospital.

The current study found that those mothers who had the lowest income (≤1000 ETB) per month were 3.99 times more likely to develop maternal near-misses as compared to their counter parts. This study is consistent with a study conducted in the Kathmandu Medical College of Teaching Hospital [24] and Muzaffarnagar, India [25]. This might be due to financial constraints that women face, leading to delays in access care, even when they have obstetric complications. The lack of economic power may make women dependent on the decision-making of others, even to access health care services.

The study also revealed that those women who stayed 7 or more days at the hospital were 2.49 times more likely to develop maternal near-misses than those who stayed less than 7 days. This finding is consistent with studies conducted in Amhara regional state hospitals [11]. This might be due to participants who had a longer hospital stay that could be at a higher risk of infection development. One contributing factor to this might be surgical site infections which commonly occur from the first few weeks after operation which might be related to the lack of aseptic technique with procedures, equipment, and inadequate postoperative care.

The study suggested that the odds of developing maternal near-miss among women who had vaginal bleeding were 2.75 times more likely to develop life-threatening complication as compared to their counter parts. This report is supported with studies conducted in Nepal [10], southeast Iran [8], and Tigray, Ethiopia [12]. The possible explanation might be that women with excessive blood loss are more susceptible to shock with treatment regimens which often required blood transfusion. Hemorrhage can occur suddenly and unexpectedly. Management of obstetric hemorrhage at facilities where there is no available blood supply and no highly trained health care workers who are skilled with management of obstetric hemorrhage may increase the morbidity of women with bleeding.

This study showed that women who had initially diagnosed with pregnancy-induced hypertension were 5.3 times more likely to develop maternal near-miss than those without pregnancy-induced hypertension. This is consistent with the study done in Kathmandu Hospital [24]; Tigray, Ethiopia [12]; Sudan [21]; and southeast Iran [8].This might be a reflection of poor management of pregnancy-induced hypertension cases in the peripheral health centers as 50.8% of them were referral cases. On the other hand, this might be due to delay in seeking appropriate treatment because of lack of proper referral system and early detection of life-threatening conditions like eclampsia.

## 5. Conclusion

The proportion of maternal near-miss was found to be high. Variables like low monthly income, seven or more days of hospital stay, vaginal bleeding, and pregnancy-induced hypertension were significantly associated factors with maternal near-miss.

### 5.1. Recommendation

Early identification and management of vaginal bleeding, pregnancy-induced hypertension, and decrement of long hospital stay are recommended to the hospital. Lastly, income generation activity needs attention by region.

### 5.2. Limitation of the Study

In this study, no follow-up was done for the women after their discharge from the hospital. This may be underestimating the magnitude of maternal near-miss.

## Figures and Tables

**Table 1:** Sociodemographic characteristics of study participants at the University of Gondar Referral Hospital, Northwest Ethiopia, June, 2019 (*N* = 303).

Variables		Frequency (*n*)	Percent (%)
Age	≤20	44	14.5
	21-34	228	75.2
	≥35	31	10.2

Religion	Orthodox	269	88.8
	Muslim	32	10.6
	Protestant	2	0.7

Residence	Urban	190	62.7
	Rural	113	37.3

Marital status	Married	297	98
	Unmarried	6	2

Maternal education	Not formal education	146	48.2
	Grades 1-8	53	17.5
	High school and more	104	34.3

Maternal occupation	Housewives	225	74.3
	Government employee	48	15.8
	Merchant	22	7.3
	Farmer	1	0.3
	Daily laborer	5	2.3

Husband occupation	Farmer	114	38.4
	Government employee	90	30.3
	Merchant	85	28.6
	Daily laborer	8	2.7

Monthly income	≤1000	100	33
	1001-2000	30	9.9
	2001-3000	33	10.9
	≥3001	140	46.2

**Table 2:** Three delay model-related characteristics of study participants at the University of Gondar Referral Hospital, Northwest Ethiopia June, 2019 (*n* = 303).

Three delay model variables		Frequency	Percent
Decision maker	Self	239	78.9
	Husband	48	15.8
	Relatives	16	5.3

Delay in making decision	Yes	48	15.8
	No	255	84.2

Did you go straight to the hospital?	Yes	277	91.4%
	No	26	8.58%

Why you were delayed to reach the hospital?	Lack of transport	16	61.53
	Lack of money	4	15.38
	Bad road construction	6	23.07

Time to reach the hospital	<1 hr	136	44.9
	≥1 hr	167	55.1

Time it take to get the care in the hospital	<30	180	59.4
	≥30	123	40.6

Type of problems you faced in the hospital	Delay in making correct diagnosis	7	2.3
	No assessment by senior health care provider	3	1.0
	Lack of supply and equipment	4	1.3
	Poor monitoring of patient	5	1.7

**Table 3:** Factors associated with maternal near-miss at the University of Gondar Referral Hospital, Northwest Ethiopia, June 2019 (*n* = 303).

Variables		Near-miss		COR (95% CI)	AOR (95% CI)
		Yes (%)	No (%)		
Residence	Urban	22 (11.6)	168 (88.4)	1	
	Rural	26 (23.0)	87 (77.0)	0.01 (1.22-4.26)	

Monthly income	≤1000	29 (29.0)	71 (71)	3.99 (1.95, 8.16)	3.99 (1.65, 9.65)^∗^
	1001-2000	2 (6.7%)	28 (93.3)	0.69 (0.15, 3.26)	0.89 (0.14, 5.57)
	2001-3000	4 (12.1)	29 (87.9)	1.34 (0.41, 4.43)	1.65 (0.41, 6.67)
	≥3001	13 (9.3)	127 (90.7)	1	1

Parity	1	18 (14.2)	109 (85.8)	1	
	2-4	23 (15.4)	126 (84.6)	1.10 (0.56, 2.15)	
	≥5	7 (25.9)	20 (74.1)	2.11 (0.87, 5.73)	

Duration of hospital stay	<7days	22 (9.3)	215 (90.7)	1	
	≥7days	26 (39.4)	40 (60.6)	6.4 (3.28, 12.30)	5.43 (2.49, 11.83)^∗∗^

Admission mode	Not referred	16 (10.7)	133 (89.3)	1	
	Referred	32 (20.8)	122 (79.2)	2.18 (1.14, 4.17)	

Reason for seeking care					
Visual disturbance	Yes	17 (50)	17 (50)	7.68 (3.56, 16.56)	
	No	31 (11.5)	238 (88.5)	1	
Severe headache	Yes	16 (42.1)	22 (57.9)	5.29 (2.52, 11.12)	
	No	32 (12.1)	233 (87.9)	1	
Vaginal bleeding	Yes	18 (29.5)	43 (70.5)	2.96 (1.51, 5.78)	2.75 (1.17, 6.47)^∗∗^
	No	30 (12.4)	212 (87.6)	1	

Initial diagnosis					
PIH	Yes	18 (42.9)	24 (57.1)	5.77 (2.81, 11.86)	5.13 (2.08, 12.63)^∗∗^
	No	30 (11.5)	231 (88.5)	1	

NB: ^∗^*p* value < 0.005 and ^∗∗^*p* < 0.05.

## Data Availability

The data will be available from the corresponding author when appropriate at the requesting time.

## Abbreviations

AOR: Adjusted odds ratio
APH: Antepartum hemorrhage
CI: Confidence interval
CS: Caesarean section
EDHS: Ethiopian demographic health survey
ETB: Ethiopian birr
GTD: Gestational troplastic disease
HCT: Hematocrit
HIV: Human immune-virus
MDG: Millennium development goal
MNM: Maternal near-miss
PID: Pelvic inflammatory disease
PIH: Pregnancy-induced hypertension
PPH: Postpartum hemorrhage
SDG: Sustainable development goal
SPSS: Statistical package for the social science
STI: Sexually transmitted infection
SVD: Spontaneous vaginal delivery
WHO: World Health Organization.
